# How Changing Signaling Volume Impacts the Importance of Away Rotations in the Otolaryngology Match

**DOI:** 10.1002/oto2.70190

**Published:** 2026-01-08

**Authors:** Maya G. Hatley, Ronald S. Wang, Emmanuel Garcia Morales, Wenqing Yang, Michele Santacatterina, Angela P. Mihalic, Max M. April

**Affiliations:** ^1^ Department of Otolaryngology–Head and Neck Surgery New York University Grossman School of Medicine New York New York USA; ^2^ Department of Pediatrics—University of Texas Southwestern Medical Center Dallas Texas USA

**Keywords:** away rotations, otolaryngology match, otolaryngology residency applications, preference signaling

## Abstract

**Objective:**

Signaling was introduced to the otolaryngology match in 2021, with 5 signals allotted to applicants in 2021, 4 in 2022, 7 in 2023, and 25 in 2024. This study investigated the modifying effect of signaling volume on the relationship between away rotations and matching in otolaryngology from 2018 to 2024.

**Study Design:**

Cross‐sectional.

**Setting:**

National survey of US medical students.

**Methods:**

We used the Texas Seeking Transparency in Application to Residency (STAR) survey responses of otolaryngology applicants from 2018 to 2024. Using multivariate logistic regression, we determined the odds of matching where away rotations were performed and how these odds varied across the pre‐volume (2018‐2020), low‐volume (2021‐2023), and high‐volume (2024) signaling eras.

**Results:**

In total, 28.3% (n = 855) of otolaryngology applicants from 2018 to 2024 completed the Texas STAR survey. Using multivariate logistic regression, adjusting for applicant characteristics, and including an interaction term between performing away rotations and signaling time period, applicants in the high‐volume signaling era were found to be significantly less likely to match at programs where away rotations were performed (odds ratio [OR]: 0.56, 95% CI: 0.33‐0.95; *P* < .05) compared to the pre‐signaling era. The same trend was seen in the low‐volume signaling era, though not statistically significant (OR: 0.76, 95% CI: 0.47‐1.22, *P* = .24). The most impactful factor on matching across all study years was performing an away rotation (OR: 12.1, 95% CI: 9.0‐16.5, *P* < .001).

**Conclusion:**

The introduction of signaling and the recent increase in signal number are associated with decreased likelihood of matching at a program where an away rotation was performed compared to the pre‐signaling era.

**Level of Evidence:**

V.

*Otolaryngology–Head & Neck Surgery* (*OHNS*) is a highly competitive specialty, with significantly more applicants each year than available residency spots.^1^, ^2^ Consequently, applicants have adapted strategies to increase their odds of matching.^3^ The average number of applications per applicant was approximately 40 in 2006, 60 in 2014, and recently estimated at 86 in 2022.^3^, ^4^, ^5^ Additionally, up to 90% of applicants report applying to and accepting interviews at programs they do not have a strong interest in, to increase their overall chance of matching.^6^ Naclerio et al reported in 2014 that the mean number of applications received per program rose from 159 in 2008 to 278 in 2014.^5^ The growing volume of applications received by programs because of these applicant strategies has led to an inefficient match system, requiring increasing time and resources from both applicants and otolaryngology programs.^7^, ^8^

Preference signaling was introduced in the 2021 match to address challenges stemming from COVID‐19 pandemic restrictions, such as the inability to conduct away rotations and in‐person interviews. Preference signaling also helps mitigate some of the challenges described above.^8^ Signaling allows applicants to reliably express interest to programs through a fixed number of signals.^9^ Additionally, signaling may spark holistic review of an applicant that a program otherwise would have overlooked.^10^, ^11^ Therefore, signaling benefits both programs and applicants by decreasing financial and logistical burdens and increasing the likelihood of a mutually desired match.^11^, ^12^

In 2021, otolaryngology applicants were first able to signal up to five programs upon submitting the ERAS® application.^8^ At this time, multiple studies reported that signaling in the ENT match was found to significantly increase the odds of receiving an interview from signaled programs.^8^, ^9^, ^10^, ^11^ It is important to note that during the 2021 and 2022 match cycles, applicants were counseled not to signal home programs and programs where they had completed away rotations, while in the 2023 match cycle, applicants were instructed not to signal their home program, but to signal programs where away rotations were completed if desired.^8^ Survey studies of otolaryngology applicants have found widespread support for the use and continuation of the signaling system.^9^, ^13^, ^14^ Since the option was introduced in the 2021 match cycle, the number of signals allotted per applicant dropped to 4 in the 2022 match, increased to 7 in the 2023 match, and finally rose to 25 for the 2024 match.^9^, ^11^, ^14^ Due to the COVID‐19 pandemic, away rotations were prohibited in the 2021 match, though some students completed away rotations virtually, and limited to one to two per applicant in the 2022 match depending on whether their institution had an otolaryngology program.^9^

Cross‐sectional outcomes from the 2024 OHNS match, the first to allow up to 25 signals, have yet to be analyzed. In a recent single‐center study of match outcomes in the context of high‐volume signaling, Yousef et al found on average that 84% of interview offers in the 2024 OHNS match came from signaled programs.^14^ In the high‐volume signaling era, applicants are advised to send preference signals to both their home institution and any programs that they perform away rotations with, if they desire to do so.

Limited by time, resources, and scheduling, applicants can typically complete up to four away rotations and may miss this opportunity at their most desired programs. Cyberski et al estimated the average cost of attendance for an away rotation in otolaryngology to be $1537, with reported costs for a single away rotation as high as $5500.^7^ Signaling offers another, potentially more accessible pathway to express interest, though it may also introduce stress due to the limited number of signals.

The objective of this study is to evaluate how the odds of matching are affected by the completion of away rotations and how these odds vary between each signaling time period. The Texas Seeking Transparency in Application to Residency (STAR) database contains self‐reported survey data from a large number of otolaryngology applicants who matched in the years 2018 to 2024, and this comprehensive data can help fill the gaps in current knowledge described above.^14^, ^15^ An analysis of this cross‐sectional data is needed to provide evidence‐based guidance to students in future match cycles on how best to apply their limited time, effort, and resources in the setting of high‐volume signaling. We hypothesize that though signaling will continue to improve the odds of matching overall, increased signaling volume will be associated with decreased odds of matching at programs where away rotations are performed due to the increased ability to communicate interest with additional programs where away rotations are not conducted.

## Materials and Methods

### Data and Sample Selection

In total, there were 3024 otolaryngology applicants in the 2018 to 2024 matches, 28.3% of whom (n = 855) completed the Texas STAR survey. These 855 applicants submitted a total of 59,431 otolaryngology applications during this time period. The data used were acquired with permission from the University of Texas Southwestern Medical Center and collected through the STAR survey. The data are de‐identified and self‐reported by participating allopathic and osteopathic medical students following the match. Students were asked to record information for each program they applied to, including whether they performed an away rotation, sent a preference signal, or had a geographic connection to that program. This study does not constitute human subject research under federal guidelines and was therefore exempt from Institutional Review Board approval.

### Variables of Interest

The primary outcome of interest in this study was the odds of matching at a program where an away rotation was completed, and how these odds varied across three signaling time periods. The primary variables of interest were the completion of away rotations, the use of signaling, and the time period. The pre‐signaling era was defined as the match cycles from 2018 to 2020, while the low‐volume signaling era was defined as the match cycles from 2021 to 2023, and the high‐volume signaling era was defined as the match cycle of 2024.

### Other Covariates

Categorical covariates included in our regression analysis included whether a participant had a geographic connection to, performed an away rotation with, and/or sent a preference signal to each program they were applying to, as well as whether the applicant was a member of the Alpha Omega Alpha Honor Medical Society (AOA), if they took an additional year (or more) for the purpose of research, their class rank (by quartile, if reported by their institution), and what score ranges their Unites States Medical Licensing Examination (USMLE) scores (step 1, when scored, and step 2) fell into. Additionally, continuous variables such as the number of peer‐reviewed publications published; number of abstracts, posters, and presentations presented; number of volunteer experiences completed; number of leadership positions held; and number of clerkships honored were also included.

### Statistical Analysis

The characteristics of the study sample were described by the signaling era. The main analysis estimated the odds of matching at any program where an away rotation was completed in the pre‐volume, low‐volume, and high‐volume signaling eras, using logistic regression models. To examine how the odds of matching changed between eras, the regression model included an interaction term between the completion of an away rotation and the signaling era. We estimated both unadjusted (completion of an away rotation, the time period, and the interaction term of these two factors) and a fully adjusted model that also included the presence of a geographic connection, use of signaling, the year the application was sent in, USMLE test scores, AOA membership, class rank, number of research products, volunteer experiences completed, leadership positions held, and clerkship grades. Additional regression models were also constructed to analyze the odds of matching in the low‐ and high‐volume signaling periods separately to acquire absolute as well as relative odds of matching based on the factors listed above. Due to changes in the otolaryngology residency application process during the COVID‐19 pandemic, a sensitivity analysis was performed to examine the data without the 2021 match cycle included in the low‐volume signaling era. Odds ratios and 95% confidence intervals are reported. All statistical analyses were two‐sided and used a significance level of *P*‐value < .05. All models were checked for goodness‐of‐fit using a Hosmer‐Lemeshow test. R v4.4.1 was used for all statistical analyses.

## Results

When discussing these results, we will refer to a match cycle by the year in which the match took place. For example, the 2020 to 2021 application cycle will be referred to as the 2021 match cycle.

In total, 855 students out of the total 3024 applicants to otolaryngology residency in the 2018 to 2024 match cycles completed the Texas STAR survey, giving a response rate of 28.3%. In total, 58,650 unique otolaryngology residency applications were submitted during this time period, yielding a mean of 68.6 applications per applicant across all years (SD: 31.9). Notably, the mean number of applications per applicants were stable in the 70s during the low‐volume signaling era (2021‐2023), then decreased to 46.8 (SD: 24.5) in 2024, the high‐volume signaling era. These and more demographic characteristics of the Texas STAR respondents are shown in detail in Table 1.

**Table 1:** Otolaryngology Applicant Responses to Texas Seeking Transparency in Application to Residency (STAR) Survey in the Signaling Era^a^

	2018	2019	2020	2021	2022	2023	2024
Number of respondents	70	105	143	115	137	146	139
Signals allotted per person	0	0	0	5	4	7	25
Applications sent per person, mean (SD)	65.5 (20.5)	65.1 (24.6)	70.3 (26.4)	73.7 (33.8)	75.4 (35.4)	78.5 (37.3)	46.9 (24.5)
Away rotations done per person, mean (SD)	1.8 (1.1)	2.2 (1.1)	2.2 (1.2)	0.29 (0.9)	1.47 (1.59)	2.5 (1.3)	2.7 (1.2)
Signals sent per applicant, mean (SD)	0 (0)	0 (0)	0 (0)	4.5 (1.5)	3.2 (1.4)	6.6 (1.7)	24.5 (4.0)
Interviews offered per applicant, mean (SD)	NA	17.4 (9.1)	17.2 (10.2)	13.6 (7.8)	12.8 (8.5)	15.3 (8.6)	13.4 (5.6)
Interviews attended per applicant, mean (SD)	14.7 (4.3)	12.3 (4.2)	12.1 (5.2)	11.9 (5.5)	11.4 (6.7)	13.2 (6.2)	11.9 (4.4)
Abstracts/posters/presentations per applicant, mean (SD)	6.6 (3.6)	7.0 (3.9)	8.2 (4.0)	8.4 (3.8)	8.8 (3.4)	9.4 (3.5)	9.4 (3.5)
Research publications per applicant, mean (SD)	3.2 (2.9)	4.0 (3.5)	5.0 (3.7)	5.3 (3.6)	5.7 (3.5)	6.7 (4.0)	4.4 (3.6)
Volunteer experiences per applicant, mean (SD)	7.3 (3.2)	6.9 (3.3)	8.1 (3.1)	7.8 (3.1)	8.0 (3.2)	7.8 (3.2)	4.0 (2.5)
Clerkships honored per applicant, mean (SD)	4.3 (2.8)	4.7 (2.3)	4.5 (2.4)	4.4 (2.4)	3.6 (2.6)	3.7 (2.5)	4.5 (2.2)
Leadership positions per applicant, mean (SD)	4.9 (3.3)	5.5 (2.6)	5.0 (3.0)	5.3 (3.1)	5.1 (3.0)	5.5 (3.1)	3.8 (2.2)

^a^ This table displays key statistical measures of responses of otolaryngology applicants to the Texas STAR survey, including the number of respondents, mean number of applications per person, mean number of signals sent to programs per person, mean number of interviews offered and attended, and percentage of applicants who matched, during the low‐ and high‐volume signaling eras.

Across all years, performing an away rotation was the factor most strongly associated with increased odds of matching at a specific program (OR: 12.15, 95% CI: 9.0‐16.5; *P* < .001), shown in Table 2. From 2021 to 2024, sending a preference signal was also found to greatly increase the odds of matching at the program where that signal was sent, also shown in Table 2 (OR: 4.31, 95% CI: 3.2‐5.8; *P* < .001). This is also noted in Table 2. The full results of the regression model for each applicant characteristic are provided in Supplemental Table S1, available online.

**Table 2:** Fully Adjusted Regression Model Estimating Odds of Matching^a^

	OR	95% CI	*P*‐value
Away rotation performed	12.1	[9.0, 16.5]	*P* < .001
Preference signal submitted	4.30	[3.2, 5.8]	*P* < .001
Geographic connection endorsed	3.67	[2.9, 4.5]	*P* < .001
Signaling period			
Low‐volume signaling period	1.28	[1.0, 1.6]	*P* = .046
High‐volume signaling period	1.12	[0.67, 1.88]	*P* = .658
Interaction term: away rotation + signaling period			
Away rotation performed + low‐volume signaling period	0.76	[0.47, 1.22]	*P* = .252
Away rotation performed + high‐volume signaling period	0.56	[0.32, 0.95]	*P* = .031

Abbreviation: OR, odds ratio.

^a^ Results are derived from multivariate regression models adjusted for the following covariates: the presence of a geographic connection, use of signaling, the year the application was sent in, Unites States Medical Licensing Examination (USMLE) test scores, Alpha Omega Alpha Honor Medical Society (AOA) membership, class rank, number of research products, volunteer experiences completed, leadership positions held, and clerkship grades. These variables were selected based on their potential to confound the relationship between the exposure and outcome. This table displays odds ratios calculated via multivariate logistic regression of matching based on application‐specific characteristics. The reference time period for the low‐volume and high‐volume signaling periods is the pre‐signaling era.

### Low‐Volume Signaling Era

Applicants to otolaryngology residency in the low‐volume signaling era (2021‐2023) were found to have significantly greater odds of matching overall compared to individuals in the pre‐signaling era (OR: 1.28, 95% CI: 1.0‐1.6, *P* < .05), shown in Table 2. Within this time period, performing an away rotation was the most significant factor influencing matching at a specific program (OR: 13.45, 95% CI: 9.5‐19.1, *P* < .001). To determine the modifying effect of the low‐volume signaling time period on the relationship between matching and performing away rotations, multivariable logistic regression analysis, including an interaction term between the signaling time period and performing an away rotation, was done as described in our statistical methods. From 2021 to 2023, the likelihood of matching at a program where an away rotation was completed was found to be lower than in the pre‐signaling era, though this difference was not statistically significant (OR: 0.76, 95% CI: 0.47‐1.22; *P* = .25), as shown in Table 2.

In a sensitivity analysis without data from 2021, the overall odds of matching in the low‐volume signaling era (2022‐2023) were not found to be significantly different from the odds of matching before signaling (OR: 1.28, 95% CI: 9.3‐1.7, *P* = .13). Additionally, the odds of matching at a program where an away rotation was completed during the low‐volume signaling era were not found to be significantly different from that of the pre‐signaling era (OR: 0.82, 95% CI: 0.49‐1.36) in this sensitivity analysis.

### High‐Volume Signaling Era

Applicants to otolaryngology residency in the high‐volume signaling era (2021‐2023) were not found to have significantly different odds of matching overall compared to individuals in the pre‐signaling era (OR: 1.12, 95% CI: 0.67‐1.88, *P* < .05) (Table 2). Yet again, within this time period, the most significant factor influencing matching at a specific program was performing an away rotation at that program (OR: 10.29, 95% CI: 6.4‐16.6, *P* < .001). The same multivariate logistic regression was also used to determine the modifying effect of the high‐volume signaling era on the relationship between matching and performing away rotations, through the interaction term described previously (Table 2). In 2024, the likelihood of matching at a program where an away rotation was completed was found to be significantly lower than in the pre‐signaling era (OR: 0.56, 95% CI: 0.33‐0.95; *P* < .05) (Table 2). In the sensitivity analysis performed, similar results were found, with applicants having lower odds of matching at a program where an away rotation was completed compared to the pre‐signaling era (OR: 0.57, 95% CI: 0.33‐0.97; *P* < .05).

### Unadjusted Regression Model

To determine the impact of each of the covariates included in our analysis on the results reported above, an unadjusted regression model was constructed and is shown in Table 3.

**Table 3:** Unadjusted Regression Model Estimating Odds of Matching^a^

	OR	95% CI	*P*‐value
Away rotation performed	18.82	[14.6, 24.3]	*P* < .001
Signaling time period			
Low‐volume signaling period	0.83	[0.72, 0.96]	*P* < .01
High‐volume signaling period	1.24	[1.00, 1.52]	*P* = .046
Interaction term: away rotation + signaling period			
Away rotation performed + low‐volume signaling	1.27	[0.88, 1.82]	*P* = .202
Away rotation performed + high‐volume signaling	0.70	[0.44, 1.14]	*P* = .153

Abbreviation: OR, odds ratio.

^a^ This table displays the unadjusted odds ratios calculated through multivariate logistic regression of matching. The reference for the low‐volume and high‐volume signaling periods is the pre‐signaling era.

## Discussion

Our results show that applicants to otolaryngology residency have a lower likelihood of matching at a program where an away rotation was performed in the high‐volume signaling era (2024) compared to the pre‐signaling era. These findings demonstrate that signaling is accomplishing its intended goal of helping applicants express interest to a wider variety of programs, and consequently, match at programs where they did not have the time or opportunity to complete an away rotation. Crucially, these findings do not suggest that away rotations are no longer impactful, but rather that they are *less* impactful on the odds of matching compared to the pre‐signaling era. As described in our results, performing an away rotation still significantly increased the odds of matching at that program in each time period, with a 13.5‐fold increase in matching odds in the low‐volume signaling era and a 10‐fold increase in matching odds in the high‐volume signaling era.

Before the introduction of signaling, away rotations were the primary method of demonstrating interest.^13^ In the high‐signaling era, away rotations are less influential on matching overall, likely because they are no longer one of the only ways to increase an applicant's odds of securing an interview or match at a *specific* program. This is an essential result to communicate to applicants to OHNS residency in the coming years, as this clarifies not only how signals are used and perceived by residency programs in the high‐volume signaling era, but also the potentially changing roles and possible uses of signals and away rotations.

Our results show that, during the low‐volume signaling period, applicants did not have different odds of matching at a program where they completed an away rotation compared to the pre‐signaling era. Though communication between applicants and programs may have been somewhat improved by signaling, the low number of signals likely did not solve the inefficiencies of the system we described. This may be due to the average number of applications sent per applicant at that time being much greater, by a factor of 10, than the number of allotted signals. As a result, programs likely continued to invest significant resources in evaluating applicants without clear insight into their interest level.^13^

High‐volume signaling may have helped address these concerns. With 25 signals available to each applicant, each signal conveys less strength of interest than before.^12^ However, not signaling a program is more likely to convey a lack of interest in that program compared to the low‐volume signaling era.^12^ Our results show that applicants were 56% less likely to match at a program where an away rotation was performed compared to the pre‐signaling era. The increased impact of signaling on matching odds shows that more signals have been effective in further improving applicants' chances of matching at programs beyond those they rotated with, a clear benefit to applicants. Additionally, high‐volume signaling may allow programs to focus primarily on applicants who signal them, streamlining resident selection based on applicant preferences.

Given the average of 78.5 applications sent per applicant in 2023, increasing the number of signals was a logical step to improve communication.^3^, ^14^, ^16^ High‐volume signaling may also impose an unofficial limit on the number of applications an applicant can submit in the future, decreasing financial and logistical burdens placed on applicants and programs during this process.^12^, ^17^ Results of the Texas STAR data from the past 4 years corroborate this theory, with the average number of applications per applicant being 73.7 in 2021, 75.4 in 2022, and 78.5 in 2023, while in 2024, the first year with high‐volume signaling, the average number of applications per applicant in otolaryngology was 46.9. Furthermore, it has been shown that this decreasing number of applications in the OHNS match translates to significant savings in cost and time for both applicants and residency programs.^18^ While based on only one year of data, this shift supports the idea that more signals can reduce the volume of applications sent.

Our results are concordant with previous literature that signaling and away rotations both strongly increase the likelihood of matching, with performing an away rotation being found to be the most influential factor in program‐specific matching across all years in our study period.^9^, ^10^, ^11^ Interestingly, rather than always simply reflecting the program a student most desires to match to, away rotations may also be strategically chosen by applicants based on factors including a program's rank or perceived difficulty to match with, the applicant's own competitiveness within the match, and the potential to unofficially “communicate” interest or openness to certain geographic areas. With high‐volume signaling, applicants are able to clearly and effectively communicate with a greater number of programs, potentially allowing applicants greater flexibility in which away rotations they choose to attend and why. Underscoring this point, most otolaryngology program directors report viewing signaling as a significant factor in identifying candidates to interview and support high‐volume signaling.^19^

One limitation of this study is that we were unable to account for the home institution of applicants in their match outcomes, as the Texas STAR data do not provide this information for the protection of applicant anonymity. Additionally, there are many other aspects of the match process that cannot be controlled for, including performance in interviews and away rotations and the specific guidance given to applicants on how to use signals during different years. This is especially significant in light of the COVID‐19 pandemic, which occurred during the low‐volume signaling period. The sensitivity analysis conducted removes data from 2021, the match cycle most impacted by pandemic restrictions (ie, a complete lack of in‐person away rotations), and shows that conclusions stay consistent with these data omitted. Other unmeasured potential confounders may include transitions between in‐person and virtual interviews; the use of virtual away rotations, especially in 2021; and the transition of step 1 scoring to pass/fail.

The incomplete response rate to the Texas STAR survey could result in bias if those responding to the survey are different from the overall population of otolaryngology applicants. To determine the extent to which the Texas STAR data may have been affected by response bias, key statistics from each year in the study period are shown from our data and compared to the data from the comprehensive statistics released by the National Resident Matching Program (NRMP) in Table 4.^20^, ^21^, ^22^, ^23^, ^24^, ^25^, ^26^ Though the number of respondents to the Texas STAR survey are clearly less than the overall number of applicants, the percentages of applicants who matched were similar for most years in the study period, increasing the generalizability of our results.

**Table 4:** Texas Seeking Transparency in Application to Residency (STAR) Survey Results Versus National Resident Matching Program (NRMP) Data^a^

Match cycle	Source	US senior applicants, n	Degree type, n		Applicants who matched, %
			MD	DO	
2018	Texas STAR	70	70	0	92.86
	NRMP	299	299	0	94.98
2019	Texas STAR	105	105	0	81.90
	NRMP	398	398	0	77.39
2020	Texas STAR	143	142	1	86.71
	NRMP	454	421	33	72.03
2021	Texas STAR	115	114	1	72.17
	NRMP	491	454	37	66.40
2022	Texas STAR	137	132	5	76.64
	NRMP	504	463	41	66.87
2023	Texas STAR	146	140	6	82.88
	NRMP	413	379	34	80.63
2024	Texas STAR	139	135	4	83.45
	NRMP	465	422	43	87.53

^a^ This table compares key match statistics between the Texas STAR survey and the National Resident Matching Program data, showing the number of applicants included in the analysis, degree type, and the percentage of applicants who matched in each analysis for each year in the study period.

## Conclusions

The introduction of signaling in the otolaryngology match has decreased the influence of away rotations on match outcomes. Signaling provides a reliable mechanism for applicants to otolaryngology to demonstrate interest and increase their likelihood of matching at specific programs. Furthermore, signal use combined with away rotation completion was associated with a significantly increased likelihood of matching, showing that these two mechanisms work well together to improve the matching process for programs and applicants alike.

## Author Contributions

**Maya G. Hatley**, conceptualiztion, statistical analysis, interpretation of statistical analyses, manuscript drafting, essential manuscript revisions; **Ronald S. Wang**, conceptualization, initial statistical analysis, manuscript drafting; **Emmanuel Garcia Morales**, revision of statistical analysis, interpretation of statistical analysis, essential manuscript revisions; **Wenqing Yang**, planning of statistical analysis, interpretation of statistical analysis, manuscript drafting; **Michele Santacatterina**, oversight of planning and execution of statistical analyses, manuscript drafting; **Angela P. Mihalic**, enabled access to and interpretation of data, methodology, manuscript revisions; **Max M. April**, supervision, conceptualization, data analysis, manusript revision and approval.

## Disclosures

### Competing interests

The authors declare no conflicts of interest.

### Funding source

No funding sources were used for this project.

## Supporting information

Supporting Information.
