# Supplementary material for: How Changing Signaling Volume Impacts the Importance of Away Rotations in the Otolaryngology Match
Source: OTO Open. 2026 Jan 8;10(1):e70190. doi: 10.1002/oto2.70190 (PMC12780956; doi:10.1002/oto2.70190)
Supplement: Supplementary file 1 — Supporting Information. [file OTO2-10-e70190-s001.docx]

Supplemental Table 1. Complete Results of Regression Model

|  | Odds Ratio | 95% Confidence Interval | | P-value |
| --- | --- | --- | --- | --- |
|  |  | Lower Bound | Upper Bound |  |
| **Away Rotation Performed** | **12.148** | **8.959** | **16.472** | **< 0.001** |
| **Geographic Connection Endorsed** | **3.668** | **2.986** | **4.504** | **< 0.001** |
| Signaling Time Period, Reference = Pre-Signaling Time Period |  |  |  |  |
| **Low-Volume Signaling** | **1.276** | **1.003** | **1.621** | **0.046** |
| High Volume Signaling | 1.124 | 0.670 | 1.885 | 0.658 |
| **Signal Used** | **4.309** | **3.210** | **5.783** | **< 0.001** |
| Step 1 Score Range, Reference = N/A |  |  |  |  |
| **205-209** | **< 0.001** | **< 0.001** | **< 0.001** | **< 0.001** |
| 210-214 | 0.579 | 0.104 | 3.239 | 0.534 |
| 215-219 | 0.660 | 0.270 | 1.614 | 0.363 |
| 220-224 | 0.892 | 0.528 | 1.507 | 0.669 |
| 225-229 | 0.975 | 0.642 | 1.480 | 0.904 |
| 230-234 | 1.309 | 0.940 | 1.823 | 0.111 |
| 235-239 | 1.126 | 0.823 | 1.539 | 0.457 |
| 240-244 | 1.164 | 0.872 | 1.552 | 0.302 |
| 245-249 | 1.218 | 0.909 | 1.632 | 0.186 |
| 250-254 | 1.248 | 0.935 | 1.666 | 0.133 |
| 255-259 | 1.137 | 0.837 | 1.543 | 0.411 |
| 260-264 | 1.246 | 0.923 | 1.682 | 0.151 |
| 265-269 | 1.326 | 0.947 | 1.858 | 0.101 |
| **270+** | **1.887** | **1.249** | **2.851** | **0.003** |
| Step 2 Score Range, Reference = N/A |  |  |  |  |
| 215-219 | 0.635 | 0.053 | 7.542 | 0.719 |
| 220-224 | 2.207 | 0.218 | 22.373 | 0.503 |
| 225-229 | 1.343 | 0.117 | 15.423 | 0.813 |
| 230-234 | 0.494 | 0.036 | 6.703 | 0.596 |
| 235-239 | 0.620 | 0.053 | 7.183 | 0.702 |
| 240-244 | 0.705 | 0.063 | 7.900 | 0.777 |
| 245-249 | 0.873 | 0.078 | 9.733 | 0.912 |
| 250-254 | 0.825 | 0.074 | 9.220 | 0.876 |
| 255-259 | 0.907 | 0.081 | 10.104 | 0.936 |
| 260-264 | 0.855 | 0.077 | 9.505 | 0.898 |
| 265-269 | 0.882 | 0.079 | 9.810 | 0.918 |
| 270+ | 0.673 | 0.060 | 7.506 | 0.748 |
| Alpha Omega Alpha Membership, Reference = Non-Member |  |  |  |  |
| No School Chapter | 0.984 | 0.842 | 1.149 | 0.836 |
| Member | 1.083 | 0.955 | 1.228 | 0.214 |
| Cumulative Class Quartile, Reference = 1st Quartile |  |  |  |  |
| 2nd Quartile | 1.031 | 0.898 | 1.183 | 0.666 |
| 3rd Quartile | 0.989 | 0.711 | 1.375 | 0.945 |
| 4th Quartile | 0.927 | 0.493 | 1.741 | 0.813 |
| N/A | 0.992 | 0.867 | 1.134 | 0.903 |
| **Additional Research Year** | **0.869** | **0.759** | **0.994** | **0.040** |
| **# of Abstracts/Posters/Presentations** | **1.024** | **1.009** | **1.040** | **0.002** |
| # of Published Peer Reviewed Publications | 1.002 | 0.987 | 1.018 | 0.748 |
| # of Volunteer Experiences | 0.985 | 0.967 | 1.003 | 0.109 |
| # of Leadership Positions | 1.003 | 0.982 | 1.024 | 0.780 |
| # of Clerkships Honored | 1.013 | 0.992 | 1.035 | 0.237 |
| Interaction Between Away Rotation and Signaling Time Period  Reference: Away Rotation + Pre-Signaling |  |  |  |  |
| Away Rotation + Low-Volume Signaling | 0.757 | 0.470 | 1.219 | 0.252 |
| **Away Rotation + High-Volume Signaling** | **0.557** | **0.327** | **0.949** | **0.031** |

This supplemental table demonstrates all results from the fully adjusted multivariate regression model, including all applicant characteristics. Reference categories for categorical values are noted, when appropriate. Significant results are presented in bold.
